# Correlations between forced oscillation technique parameters and pulmonary densitovolumetry values in patients with acromegaly

**DOI:** 10.1590/1414-431X20154786

**Published:** 2015-08-04

**Authors:** G.B. Camilo, A.R.S. Carvalho, D.C. Machado, R. Mogami, L. Kasuki, M.R. Gadelha, P.L. Melo, A.J. Lopes

**Affiliations:** 1 Programa de Pós-Graduação em Ciências Médicas, Faculdade de Ciências Médicas, Universidade do Estado do Rio de Janeiro, Rio de Janeiro, RJ, Brasil; 2 Departamento de Radiologia, Universidade do Estado do Rio de Janeiro, Rio de Janeiro, RJ, Brasil; 3 Instituto Estadual do Cérebro Paulo Niemeyer, Rio de Janeiro, RJ, Brasil; 4 Hospital e Maternidade Therezinha de Jesus, Faculdade de Ciências Médicas e da Saúde de Juiz de Fora, Juiz de Fora, MG, Brasil; 5 Laboratório de Fisiologia da Respiração, Instituto de Biofísica Carlos Chagas Filho, Universidade Federal do Rio de Janeiro, Rio de Janeiro, RJ, Brasil; 6 Laboratório de Engenharia Pulmonar, Programa de Engenharia Biomédica, Instituto Alberto Luiz Coimbra de Pós-Graduação e Pesquisa em Engenharia, Universidade Federal do Rio de Janeiro, Rio de Janeiro, RJ, Brasil; 7 Departmento de Endocrinologia, Hospital Universitário Clementino Fraga Filho, Universidade Federal do Rio de Janeiro, Rio de Janeiro, RJ, Brasil; 8 Laboratório de Instrumentação Biomédica, Instituto de Biologia, Faculdade de Engenharia, Universidade do Estado do Rio de Janeiro, Rio de Janeiro, RJ, Brasil; 9 Programa de Pós-Graduação em Fisiopatologia Clínica e Experimental, Faculdade de Ciências Médicas, Universidade do Estado do Rio de Janeiro, Rio de Janeiro, RJ, Brasil; 10 Laboratório de Função Pulmonar, Hospital Universitário Pedro Ernesto, Universidade do Estado do Rio de Janeiro, Rio de Janeiro, RJ, Brasil

**Keywords:** Acromegaly, Tomography, Respiratory function tests, Forced oscillation technique

## Abstract

The aims of this study were to evaluate the forced oscillation technique (FOT) and pulmonary densitovolumetry in acromegalic patients and to examine the correlations between these findings. In this cross-sectional study, 29 non-smoking acromegalic patients and 17 paired controls were subjected to the FOT and quantification of lung volume using multidetector computed tomography (Q-MDCT). Compared with the controls, the acromegalic patients had a higher value for resonance frequency [15.3 (10.9-19.7) *vs* 11.4 (9.05-17.6) Hz, P=0.023] and a lower value for mean reactance [0.32 (0.21-0.64) *vs* 0.49 (0.34-0.96) cm H_2_O/L/s^2^, P=0.005]. In inspiratory Q-MDCT, the acromegalic patients had higher percentages of total lung volume (TLV) for nonaerated and poorly aerated areas [0.42% (0.30-0.51%) *vs* 0.25% (0.20-0.32%), P=0.039 and 3.25% (2.48-3.46%) *vs* 1.70% (1.45-2.15%), P=0.001, respectively]. Furthermore, the acromegalic patients had higher values for total lung mass in both inspiratory and expiratory Q-MDCT [821 (635-923) *vs* 696 (599-769) g, P=0.021 and 844 (650-945) *vs* 637 (536-736) g, P=0.009, respectively]. In inspiratory Q-MDCT, TLV showed significant correlations with all FOT parameters. The TLV of hyperaerated areas showed significant correlations with intercept resistance (r_s_=−0.602, P<0.001) and mean resistance (r_s_=−0.580, P<0.001). These data showed that acromegalic patients have increased amounts of lung tissue as well as nonaerated and poorly aerated areas. Functionally, there was a loss of homogeneity of the respiratory system. Moreover, there were correlations between the structural and functional findings of the respiratory system, consistent with the pathophysiology of the disease.

## Introduction

Excessive production of growth hormone (GH) and insulin-like growth factor (IGF) results in the development of acromegaly. This multisystem disease usually begins between 25 and 40 years of age and occurs at similar rates among men and women (1-3). Its presentation is nonspecific, and its prevalence ranges from 40 to 130 cases per million adults (2-4). Respiratory problems are believed to account for approximately 25% of all deaths among patients with acromegaly (4-6). These patients develop a variety of respiratory disorders that affect muscle activity, cartilage and bone structure, and lung volume. More recently, changes in the lung parenchyma of acromegalic patients have also been described (4,7).

Some methods for measuring lung function have gained importance with the aim of better understanding of pulmonary complications. Such methods include the forced oscillation technique (FOT), which is potentially important because it has characteristics that are complementary to classical techniques and can provide a more detailed examination, especially considering its ability to provide early detection of abnormalities in pulmonary function (8-10). Moreover, the FOT is a simple method for investigating the mechanical properties of the respiratory system and represents the current state of the art in the assessment of lung function (11). This method characterizes the respiratory impedance and its two components, respiratory system resistance and reactance. To the best of our knowledge, no previous studies have evaluated the FOT in acromegalic patients.

Early diagnosis of lung complications can be effectively achieved using computed tomography (CT), which allows the diagnosis of complications caused by various systemic diseases (12,13). Impairment of the pulmonary parenchyma in acromegaly was reported in a previous study (7). In a more advanced manner, lung volume can be assessed using volumetric imaging techniques such as multidetector computed tomography (MDCT) (14). Image processing for quantification of lung volume using MDCT (Q-MDCT) is accurate in predicting lung function and enables examination of correlations with other classical methods of volumetric analysis. Furthermore, Q-MDCT has the potential advantage of being able to estimate regional volumes of various compartments that are currently determined using classifications previously proposed in the literature (13,15-18).

Using Q-MDCT, it is possible to assess the distribution of pulmonary aeration, calculate the volume of distinct lung regions, and analyze various lung compartments using three-dimensional histograms. We believe that both Q-MDCT and the FOT can be useful in the assessment of pulmonary impairment in acromegalic patients. We hypothesized that acromegalic patients have heavier and less-ventilated lungs, and that there is a correlation between lung structure and function in these patients. This study aimed to evaluate the use of pulmonary densitovolumetry and the FOT in acromegalic patients and to examine the correlations between these findings.

## Patients and Methods

### Patients

This cross-sectional study was conducted between June 2012 and July 2014 and evaluated 37 patients aged >18 years presenting with acromegaly. Patients who regularly attended the Hospital Universitário Clementino Fraga Filho of the Universidade Federal do Rio de Janeiro, and had a diagnosis of acromegaly suspected by clinical characteristics and confirmed by high levels of unsuppressed GH that did not fall to <0.4 ng/mL after an oral glucose tolerance test or IGF levels above the upper limit of the normal interval (19,20) were included. Patients with a history of smoking and those with comorbidities not related to acromegaly that could interfere with the FOT results were excluded from the study. Patients with untreated hypothyroidism and hypocortisolism were also excluded. In addition, a control group of 17 subjects (11 women) who met the inclusion and exclusion criteria and who had undergone chest CT in the Hospital Universitário Pedro Ernesto (HUPE) of the Universidade do Estado do Rio de Janeiro for any reason were asked to perform the FOT. All participants signed an informed consent form, and the study was approved by the Research Ethics Committee of the HUPE-UERJ (protocol number 234.362).

### Forced oscillation technique

The instrumentation used for the FOT was developed in the Laboratório de Instrumentação Biomédica, Universidade do Estado do Rio de Janeiro, and was described in a previous report (9). The pressure input was measured using a Honeywell 176 PC pressure transducer (Microswitch, USA), and the airway flows were measured using a screen pneumotachometer coupled to a similar transducer with a matched frequency response. To perform the FOT, the subjects remained seated, kept their heads in the neutral position, and breathed using their residual functional capacity through a tube. During the measurements, the subjects were asked to firmly support their cheeks and mouth floor using both hands, and a nose clip was used. A minimum coherence function of 0.9 was considered adequate (9). Three measurements were taken, and the final test result was the median of the three measurements.

Classical FOT parameters were used in data interpretation. Analysis of linear regression in the resistive component of the impedance in the frequency range of 4-16 Hz was used to determine the intercept resistance (R0) (21). The mean resistance (Rm), which is related to the airway caliber, was calculated using an oscillation frequency of 4-16 Hz (8). The slope of the resistive component of the respiratory impedance (S) was also obtained. The resonance frequency (Fr) was defined as the frequency at which the reactance equaled zero (22). The mean reactance (Xm) was calculated based on the entire examined frequency range (4-32 Hz). The dynamic compliance of the respiratory system (Cdyn) was estimated based on the reactance (Xrs) at 4 Hz using the equation Cdyn =−1/(2πfXrs), where f is the frequency (23). Consequently, we evaluated both the resistive properties (R0, Rm, S) and the reactive properties (Fr, Xm, Cdyn) of the respiratory system. We also estimated the impedance module at 4 Hz (Zrs4Hz), which reflects the total mechanical load of the respiratory system.

The parameters S, Fr, and Xm reflect both spatial and temporal ventilatory non-homogeneity (21,24). While S is associated with non-homogeneity in terms of resistance distribution, Fr and Xm describe non-homogeneity in terms of the reactive properties of the respiratory system (22,23).

### Pulmonary densitovolumetry

The CT images were acquired using a helical CT with 64 channels (Brilliance 40; Philips Medical Systems, USA). The reading time was set to 4 s, with an X-ray tube current of 458 mA and a voltage of 120 kVp. Each acquired image consisted of a 2-mm-thick block in cross-sections separated by a distance of 1 mm. The images were represented by a square matrix of 768 rows and 768 columns. For all participants, images were obtained during both inspiration and expiration. Iodinated contrast medium was not used in any examination.

The next step involved segmentation of the lung parenchyma. Images were exported in accordance with the instructions for DICOMDir files using CT-Processing software written in Matlab (MathWorks, USA) at the Laboratório de Fisiologia da Respiração, Instituto de Biofísica Carlos Chagas Filho, Universidade Federal do Rio de Janeiro, and pulmonary densitovolumetry was performed at the end of the process.

The total lung volume (TLV), total air volume (TAV), and total lung mass (TLM) were calculated using only the image from the region of interest (lung parenchyma). The TLV (i.e., sum of air plus tissue volume) was calculated as follows: [(size of pixel)^2^ × slice thickness × total number of pixels in region of interest for the whole lung]. The weight of the lungs was calculated as follows: [(1-VD/−1000] × (size of pixel)^2^ × slice thickness × total number of pixels in region of interest for the whole lung], where VD means the voxel density (15,17). To assess the frequency distribution of the voxels as a function of density in Hounsfield units (HU), the distribution of aeration was calculated, and a histogram of the voxel distribution was partitioned between −1000 and +100 HU. The number of voxels in each compartment was analyzed as a fraction of the number of voxels contained in the region of interest. The images were analyzed to calculate the percentages of hyperaerated (−1000 to −900 HU), normally aerated (−900 to −500 HU), poorly aerated (−500 to −100 HU), and nonaerated (−100 to +100 HU) compartments in TLV (15,17).

### Data analysis

To check the homogeneity of the sample, the Kolmogorov-Smirnov test was used. If a meaningful number of variables did not have a normal distribution, nonparametric tests were selected. The results are reported as the median and interquartile range or frequency (percentage). Numerical variables and categorical variables were compared using the Mann-Whitney test and Fisher’s exact test, respectively. Spearman's rank correlation coefficient was calculated to investigate associations. Using data with logarithmic transformation, graphics with standardized residuals were created to assess the heteroscedasticity. Data analyses were performed using the SAS 6.11 software (SAS Institute Inc., USA). The statistical significance level was set at P<0.05.

## Results

Of the 37 patients with acromegaly initially recruited, eight were excluded because of refusal to participate in the study (6 patients) or inability to reach the eligibility criteria in the FOT (2 patients). Consequently, the acromegalic group included 29 patients (19 women, 10 men) with a mean age of 46 (31-56) years. Three patients had hypopituitarism, but were undergoing hormone replacement therapy and had normal hormone levels. The acromegalic patients did not differ from the control subjects with respect to age, gender, weight, height, and body mass index (P<0.05). The general characteristics of the acromegalic patients and control subjects are outlined in Table 1.

**Table:**
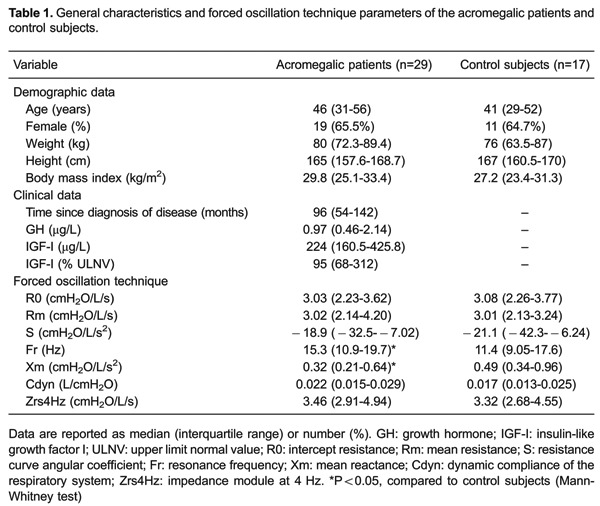

Compared with the control subjects for the FOT, the acromegalic patients had higher Fr [15.3 (10.9-19.7) *vs* 11.4 (9.05-17.6) Hz, P=0.023] and lower Xm [0.32 (0.21-0.64) *vs* 0.49 (0.34-0.96) cmH_2_O/L/s^2^, P=0.005; Table 1]. However, there were no significant differences in the FOT parameters between patients with active disease and patients with controlled disease.

Table 2 shows comparisons of the inspiratory and expiratory Q-MDCT findings between the acromegalic patients and the control subjects. With regard to inspiratory Q-MDCT, the acromegalic patients had a greater volume for poorly aerated areas [135 (118-161) *vs* 83 (65-91) mL, P=0.0001] and higher percentages of TLV for nonaerated and poorly aerated areas [0.42% (0.30-0.51%) *vs* 0.25% (0.20-0.32%), P=0.039 and 3.25% (2.48-3.46%) *vs*1.70% (1.45-2.15%), P=0.001, respectively]. For expiratory Q-MDCT, the acromegalic patients had a greater volume and a higher percentage of TLV for nonaerated areas [47.3 (37.4-66.1) *vs* 10.2 (8.31-11.7) mL, P=0.0006 and 2.15% (1.61-2.38%) *vs* 0.52% (0.31-0.63%), P=0.007, respectively]. The acromegalic patients also had higher TLM values in both inspiratory and expiratory Q-MDCT [821 (635-923) *vs*696 (599-769) g, P=0.021 and 844 (650-945) *vs* 637 (536-736) g, P=0.009, respectively].

**Table:**
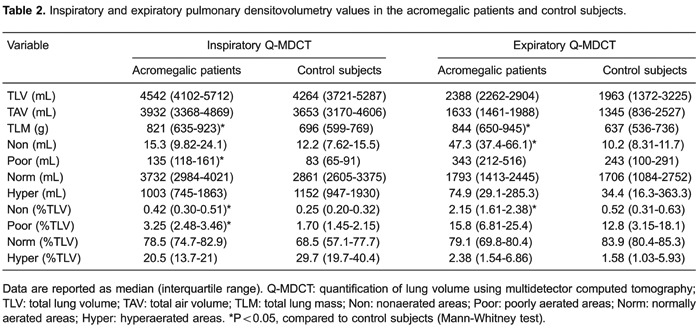

**Figure 1:**
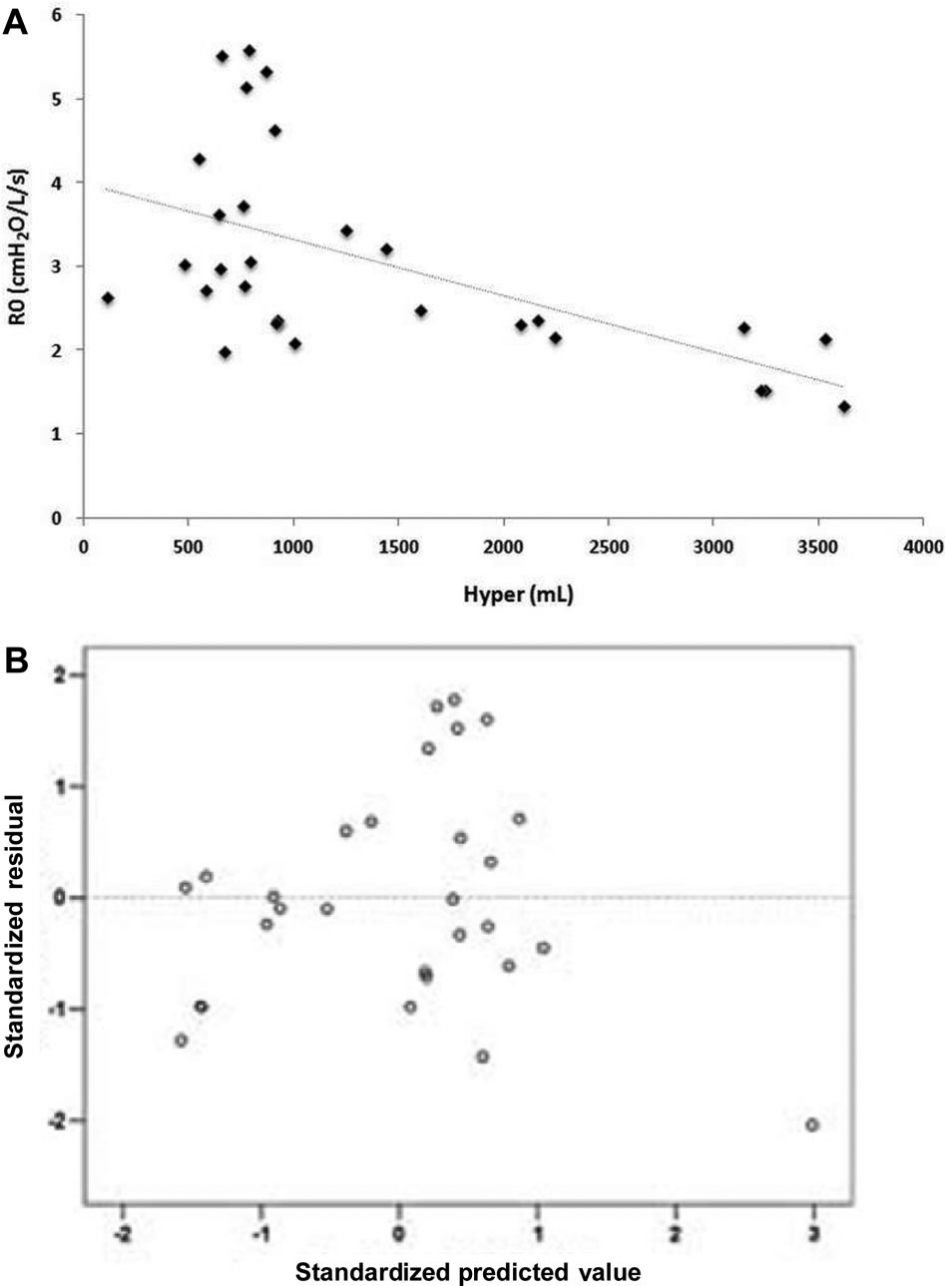
*A*, Correlation between the volume of hyperaerated areas (Hyper) and the intercept resistance (R0; r_s_=−0.602, P<0.001). *B*, Standardized residuals versus predicted values for the correlation shown in *panel A*.

**Figure 2:**
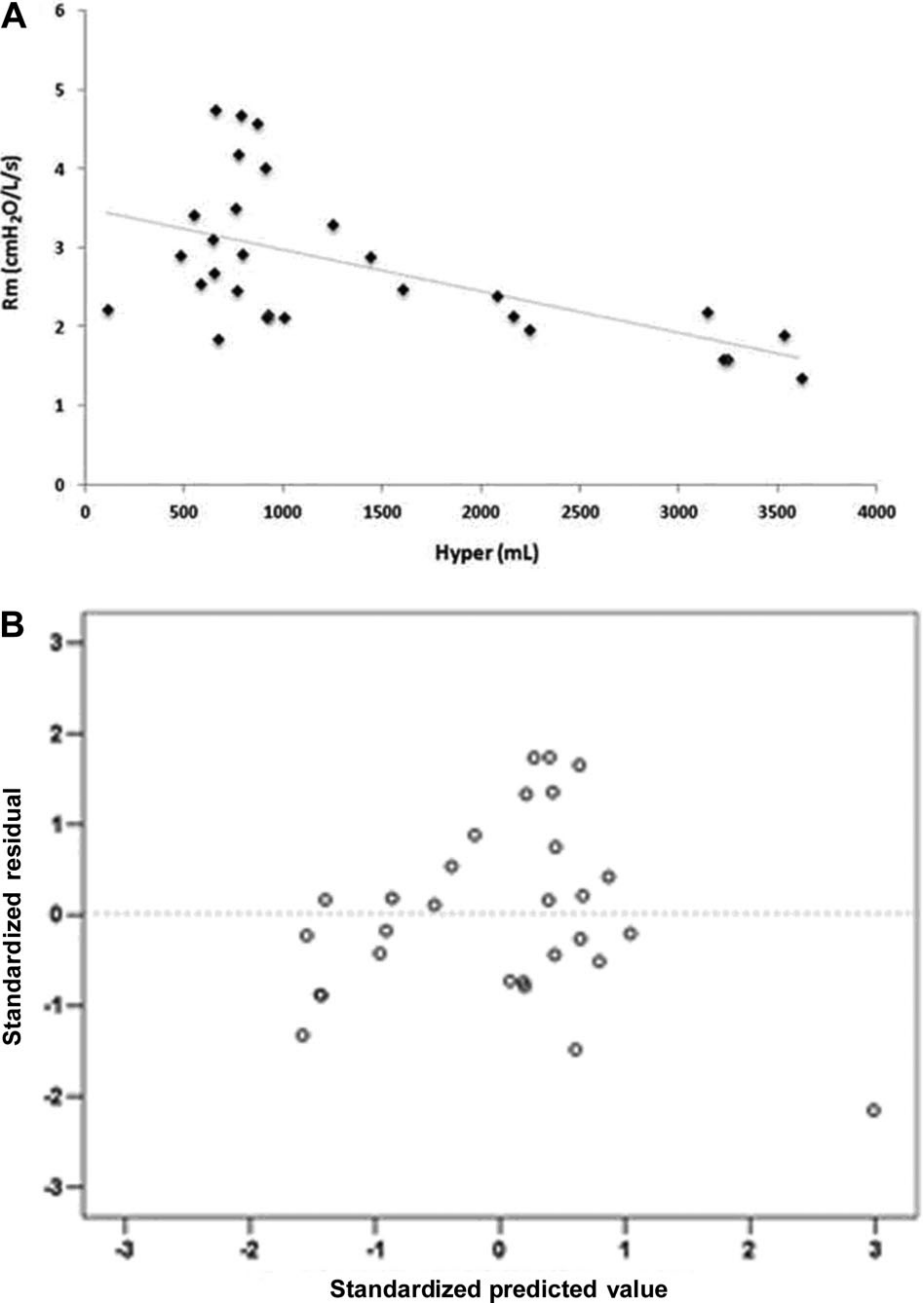
*A*, Correlation between the volume of hyperaerated areas (Hyper) and the mean resistance (Rm; r_s_=−0.580, P<0.001). *B,* Standardized residuals versus predicted values for the correlation shown in *panel A*.

**Figure 3:**
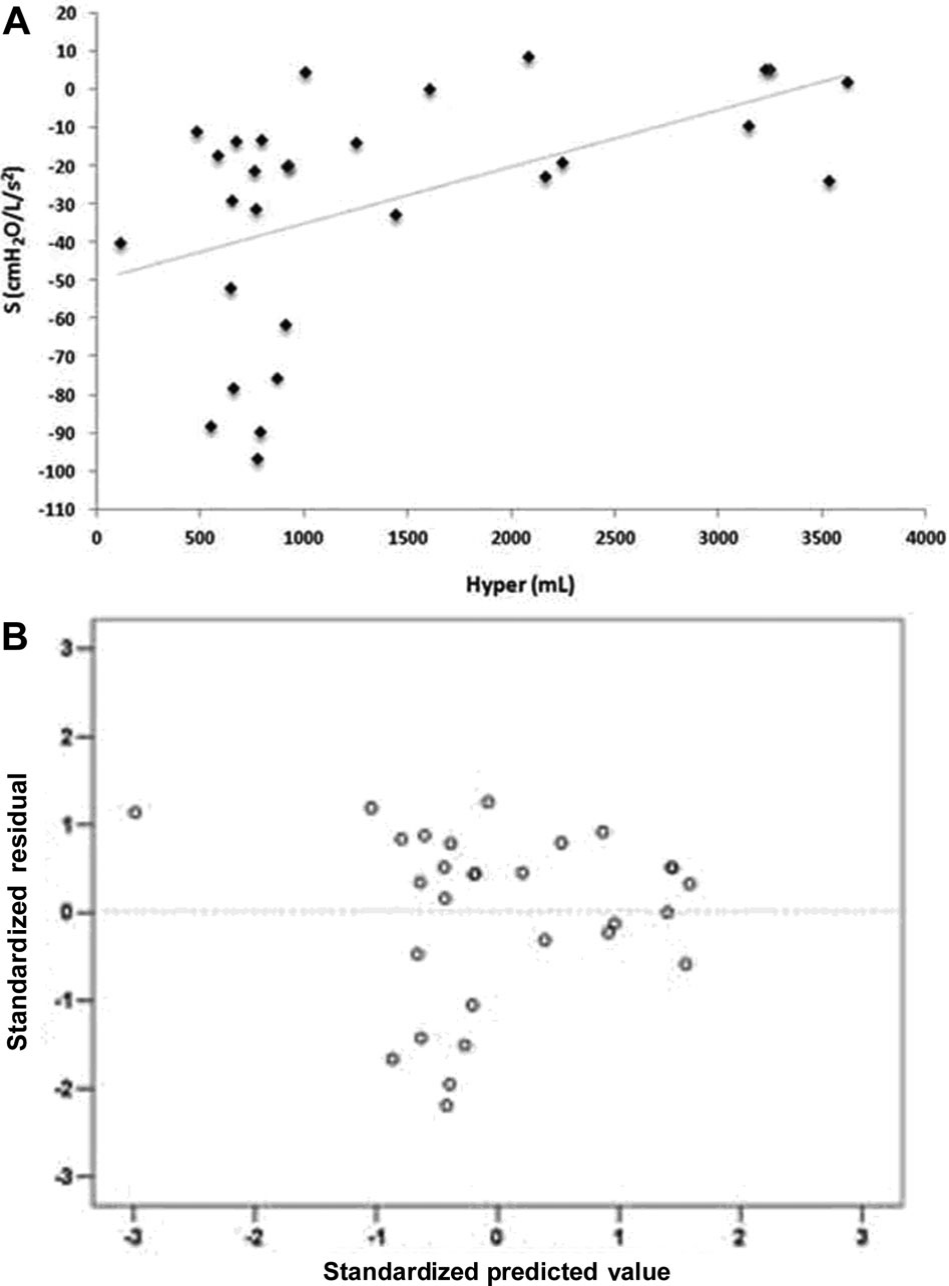
*A*, Correlation between the volume of hyperaerated areas (Hyper) and the resistance curve angular coefficient (S; r_s_=0.520, P<0.005). *B*, Standardized residuals versus predicted values for the correlation shown in *panel A.*

**Figure 4:**
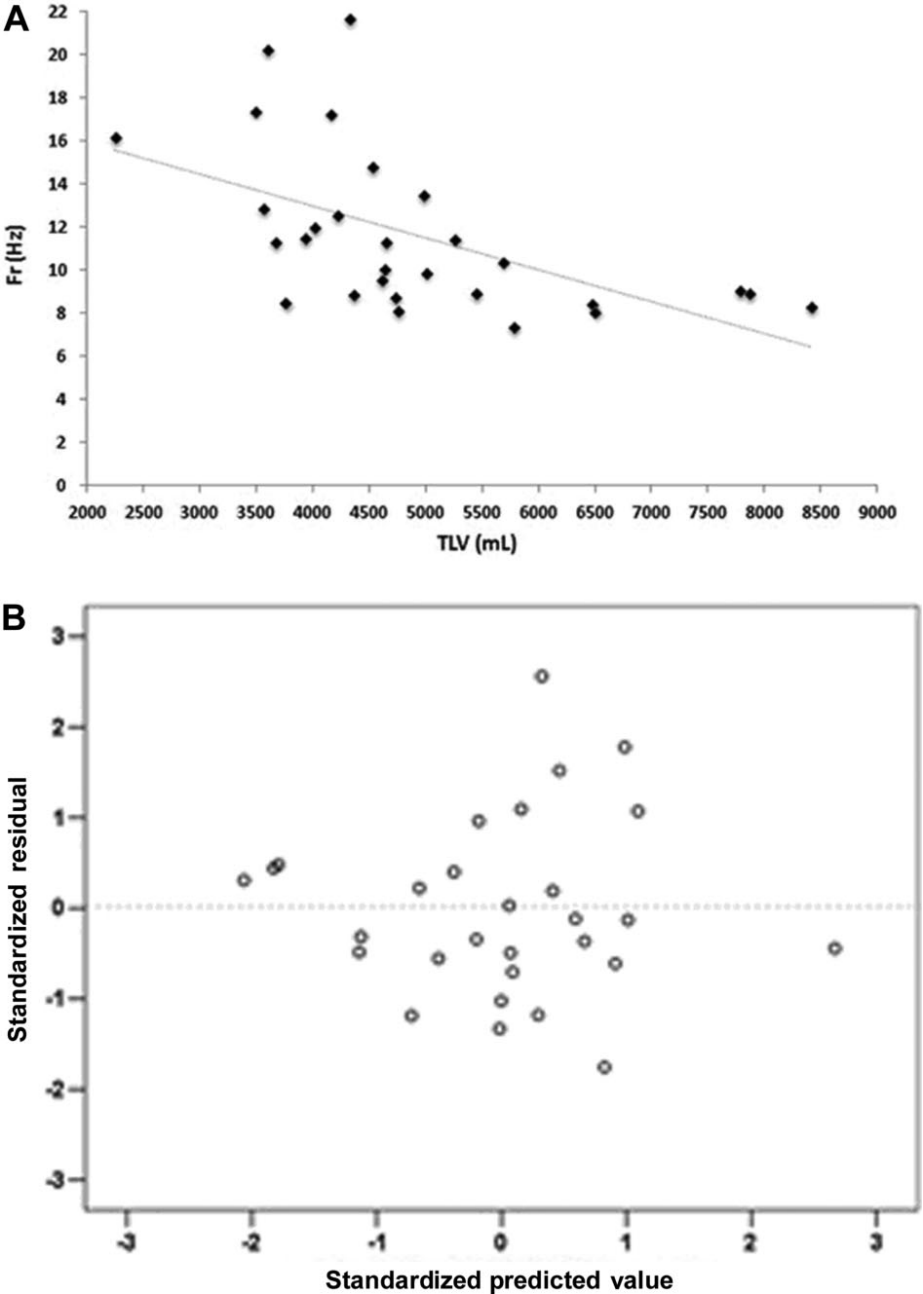
*A*, Correlation between the total lung volume (TLV) and the resonance frequency (Fr; r_s_=−0.669, P<0.001). *B,* Standardized residuals versus predicted values for the correlation shown in *panel A*.

We evaluated the correlations between the Q-MDCT findings and the FOT parameters in the acromegalic patients (Tables 3 and 4; Figures 1-4). In inspiratory Q-MDCT, both TLV and TAV showed significant correlations with all FOT parameters. The volumes of nonaerated areas and poorly aerated areas showed significant correlations with Fr (r_s_=−0.623, P<0.001 and r_s_=−0.501, P<0.01, respectively). The volume of normally aerated areas showed significant correlations with Fr (r_s_=−0.533, P<0.005) and Xm (r_s_=0.530, P<0.005). The volume of hyperaerated areas showed significant correlations with R0 (r_s_=−0.602, P<0.001), Rm (r_s_=−0.580, P<0.001), S (r_s_=0.520, P<0.005), Fr (r_s_=−0.401, P<0.05), Cdyn (r_s_=0.434, P<0.05), and Zrs4Hz (r_s_=−0.513, P<0.005). In expiratory Q-MDCT, TLV showed significant correlations with R0 (r_s_=−0.401, P<0.05), Fr (r_s_=−0.379, P<0.05), and Cdyn (r_s_=0.400, P<0.05). The volume of hyperaerated areas showed a significant correlation with Cdyn (r_s_=0.395, P<0.05).

**Table:**
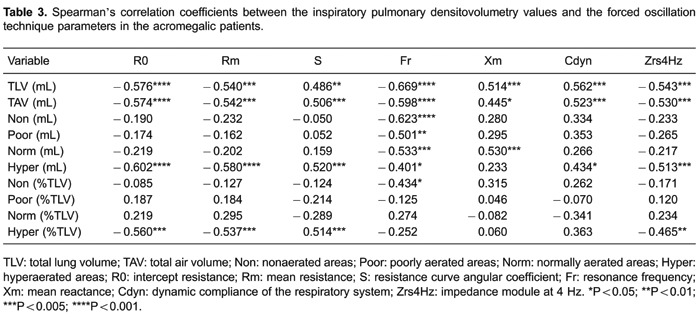

**Table:**
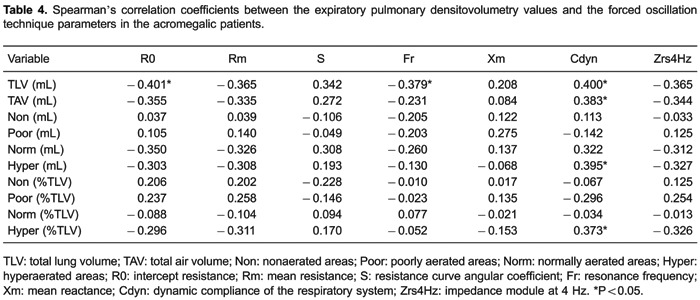

## Discussion

The main findings of this study were as follows: a) acromegalic patients experienced changes in the reactive properties of the respiratory system; b) acromegalic patients had higher amounts of nonaerated and poorly aerated areas in the lung parenchyma and increased amounts of lung tissue; and c) there were significant correlations between pulmonary densitovolumetry values and FOT parameters in acromegalic patients.

The FOT requires passive cooperation from patients and, by providing new parameters for the analysis of ventilatory mechanics, presents characteristics that are complementary to the classical methods for lung evaluation (9). Using the FOT, we observed that the acromegalic patients had higher Fr values and lower Xm values than the control subjects. These findings reflect the elastic and inertial properties of the respiratory system and indicate non-homogeneity of the respiratory system, with Fr and Xm being inversely correlated (25). The impaired lung function in acromegalic patients has not been fully elucidated, but is known to cause multiple changes that involve the bone and muscle structure of the chest and lung elasticity (4). Acromegaly alters the structure, elasticity, and function of the entire respiratory apparatus. Consequently, the heterogeneity of the respiratory system in acromegaly may result from a combination of factors, including increased pulmonary distensibility and increased transpulmonary pressure (26). Subclinical hypoxemia and ventilation-perfusion imbalance may also be involved in this process (27).

In recent years, considerable efforts have been made to use regional lung density measurements from Q-MDCT because this technique accurately reflects the air and non-air contents of the lungs and is correlated with histopathology (28). The present study showed that the acromegalic patients had heavier lungs than the control subjects. This trend was observed in both inspiratory and expiratory Q-MDCT. In this respect, there may be direct effects of GH and IGF on lung tissue, resulting in increased weight. In line with our findings, histopathological studies have shown that GH can increase the synthesis of collagen type I fibers and mucopolysaccharides in acromegalic patients (29). Interestingly, Lobie et al. (30) and Zogopoulos et al. (31) showed that GH and IGF have proliferative effects on lung cells and smooth muscle cells through interactions with their cognate receptors.

This study also showed that the acromegalic patients had greater volumes of nonaerated and poorly aerated lung areas than the control subjects. In HU units, these areas may represent areas of pulmonary consolidation and ground-glass opacities. Some authors have reported pulmonary parenchymal abnormalities in acromegalic patients observed by high-resolution CT, including increased attenuation and linear and nodular opacities (7,32). These lesions may have contributed, at least in part, to the differences in the densitovolumetry values observed in the present study. To date, no previous studies have assessed the distribution of lung aeration using Q-MDCT in acromegalic patients.

The FOT tests allowed us to obtain additional information on parameters associated with the structural properties of the respiratory system assessed by pulmonary densitovolumetry. We found that the subjects with higher TLV and TAV values exhibited greater homogeneity (positive correlations with S and Xm and negative correlation with Fr), lower elastance (positive correlation with Cdyn), and lower breathing effort (negative correlation with Zrs4Hz). Cdyn reflects the combined effects of the pulmonary and chest wall tissue, as well as the compliance of the airways (21,33). Using the esophageal balloon method, García-Río et al. (26) found decreased lung compliance in acromegalic patients after suppression of GH hypersecretion. Thus, because the FOT involves noninvasive measurements, Cdyn may be useful as a functional parameter for monitoring acromegaly, representing an alternative to the use of esophageal balloons.

It has been shown that the increased lung volume in acromegalic patients is not associated with hyperinflation or muscle strength (34). Instead, pulmonary compliance usually increases while the lung elastic recoil remains normal (35). Based on this evidence, many researchers have attributed lung overgrowth to increased alveolar size rather than an increased number of alveoli (26,36). In line with these findings, we observed that in inspiratory Q-MDCT, but not expiratory Q-MDCT, the hyperaerated areas were negatively correlated with R0 and Rm and positively correlated with S. This pattern suggests that hyperaerated areas have low resistance to airflow and high homogeneity, unlike areas of emphysema or air entrapment. Therefore, we believe that the hyperaerated areas result from the excess GH in acromegalic patients, which induces lung overgrowth by triggering an increase in alveolar size (34).

As shown in Figures 1-3, there were greater differences between the observed response variables and the estimated response variables (variability of residuals) at low values of the hyperaerated areas. In acromegalic patients, we consider that there is a progressive loss of complexity of the respiratory system as the system becomes more homogeneous with lower respiratory impedance signals. Goldberger (37) proposed that the complexity becomes lost in the presence of illness with increasing regularity of signals. According to this concept, we hypothesized that the greater variability of R0, Rm, and S for low values of the hyperaerated areas shows that these areas still have a high complexity. Since complex behaviors are best evaluated using nonlinear dynamic techniques (38), we suggest that future research should evaluate the "acromegalic lung" through nonlinear statistical models.

The strength of this study is its demonstration that the findings provided by both the FOT and pulmonary densitovolumetry are strongly correlated with the pathophysiology of acromegaly, thereby increasing the possibility of incorporating these techniques into clinical practice. We consider the small sample size to be the main limitation of our study. We also believe that performing histopathology to elucidate the pulmonary microarchitecture could reinforce the findings of the present study. Our results warrant further research on the use of the FOT and pulmonary densitovolumetry in acromegalic patients because these techniques are important instruments for monitoring such patients.

In conclusion, the present study has shown that acromegalic patients have increased amounts of lung tissue as well as nonaerated and poorly aerated areas evaluated by pulmonary densitovolumetry. These patients experience a loss of homogeneity of the respiratory system, as assessed by the FOT. Moreover, we found a correlation between lung structure and function, which is consistent with the pathophysiology of the disease.
